# Exercise Modifies the Transcriptional Regulatory Features of Monocytes in Alzheimer’s Patients: A Multi-Omics Integration Analysis Based on Single Cell Technology

**DOI:** 10.3389/fnagi.2022.881488

**Published:** 2022-05-03

**Authors:** Yisheng Chen, Yaying Sun, Zhiwen Luo, Xiangjun Chen, Yi Wang, Beijie Qi, Jinrong Lin, Wei-Wei Lin, Chenyu Sun, Yifan Zhou, Jiebin Huang, Yuzhen Xu, Jiwu Chen, Shiyi Chen

**Affiliations:** ^1^Huashan Hospital, Fudan University, Shanghai, China; ^2^Department of Neurosurgery, The Second Affiliated Hospital of Zhejiang University School of Medicine, Zhejiang University, Hangzhou, China; ^3^AMITA Health Saint Joseph Hospital Chicago, Chicago, IL, United States; ^4^Department of Ophthalmology, Shanghai General Hospital, Shanghai Jiao Tong University School of Medicine, Shanghai, China; ^5^Department of Ophthalmology, Putuo People’ s Hospital, Tongji University, Shanghai, China; ^6^Department of Pediatrics, Ruijin Hospital, Shanghai Jiao Tong University School of Medicine, Shanghai, China; ^7^Department of Rehabilitation, The Second Affiliated Hospital of Shandong First Medical University, Taian, China; ^8^Department of Orthopedics, Shanghai General Hospital, Shanghai Jiao Tong University School of Medicine, Shanghai Jiao Tong University, Shanghai, China

**Keywords:** Alzheimer’s disease, exercise, monocyte, transcription factors, cell communication

## Abstract

Monocytes have been reported to be important mediators of the protective effect of exercise against the development of Alzheimer’s disease (AD). This study aims explored the mechanism by which monocytes achieve this. Using single cell transcriptome analysis, results showed that CD14 + and CD16 + monocytes interacted with other cells in the circulating blood. *TNF*, *CCR1*, *APP*, and *AREG*, the key ligand-receptor-related genes, were found to be differentially expressed between exercise-treated and AD patients. The SCENIC analysis was performed to identify individual clusters of the key transcription factors (TFs). Nine clusters (M1-M9) were obtained from the co-expression network. Among the identified TFs, *MAFB*, *HES4*, and *FOSL1* were found to be differentially expressed in AD. Moreover, the M4 cluster to which *MAFB*, *HES4*, and *FOSL1* belonged was defined as the signature cluster for AD phenotype. Differential analysis by bulkRNA-seq revealed that the expression of *TNF*, *CCR1*, and *APP* were all upregulated after exercise (*p* < 0.05). And *ATF3*, *MAFB*, *HES4*, and *KLF4* that were identified in M4 clusters may be the TFs that regulate *TNF*, *CCR1*, and *APP* in exercise prescription. After that, *APP*, *CCR1*, *TNF*, *ATF3*, *KLF4*, *HES4*, and *MAFB* formed a regulatory network in the ERADMT gene set, and all of them were mechanistically linked. The ERADMT gene set has been found to be a potential risk marker for the development of AD and can be used as an indicator of compliance to exercise therapy in AD patients. Using single-cell integration analysis, a network of exercise-regulating TFs in monocytes was constructed for AD disease. The constructed network reveals the mechanism by which exercise regulated monocytes to confer therapeutic benefits against AD and its complications. However, this study, as a bioinformatic research, requires further experimental validation.

## Introduction

Alzheimer’s disease (AD) is a degenerative disease of the central nervous system characterized by progressive cognitive dysfunction and behavioral impairment. The disease is common in aged individuals and accounts for about 50–70% of all cases of dementia. Patients with AD present with progressive memory impairment, decreased ability to perform daily living activities, and abnormal mental behavior. Therefore, it imposes a heavy burden on society and families, making it the largest global public health issue facing humanity (Pfeffer et al., 1987). In terms of pathogenesis, it is thought to be caused by extracellular amyloid β-protein (Aβ) deposition and tau hyperphosphorylation. However, the exact pathogenesis of the disease is unknown, and there is no effective treatment available (Lei et al., 2021). The immune profile of peripheral blood has been found to be closely associated with the development of AD (Mancuso et al., 2013; Noble et al., 2014). Some pathogens (*Chlamydia pneumoniae*) may enter the brain with infected mononuclear cells (MacIntyre et al., 2003). Pathogenic invasion can trigger the increase in Aβ, thus causing long-term chronic inflammation, thus leading to AD development (Miklossy, 2011). Therefore, targeting monocytes may be a promising therapeutic approach for AD (Dobri et al., 2021).

The role of physical activity in AD prevention has received much attention in recent years (Macpherson et al., 2017; Kivipelto et al., 2018; López-Ortiz et al., 2021). However, the specific mechanisms by which exercise prevents the onset of AD should be further explored. Physical exercise can influence the transcriptome characteristics of monocytes (Strohacker et al., 2012). For instance, moderate exercise increases monocyte adhesion, molecule expression, and oxidized low-density lipoprotein (ox-LDL)-induced transendothelial migration. Monocytes are then stimulated to migrate through the endothelium (Wang et al., 2005) and thus may act as a regulator of AD prevention through exercise. Therefore, studying the effect of exercise on the transcriptional characteristics of monocytes can provide insights into the potential mechanisms of AD prevention through exercise.

Single-cellomics is a rapidly growing discipline based on single-cell sequencing, a novel high-throughput sequencing technology. Single-cell sequencing has various unique advantages in various areas, including early disease diagnosis, biological marker detection and prognosis prediction. For example, scRNA-seq analysis showed that nucleated cells in the blood play a key role in preventing AD development (Xu and Jia, 2021). Besides uncovering monocytes and their subpopulations involved in AD pathophysiology, single cell sequencing can also disclose their distinct transcriptional profiles and presumably unique functional qualities (Khan and Kaihara, 2019; Masuda et al., 2020; Tuzlak et al., 2021). Single cell sequencing can be used to elucidate the pathogenesis and identify novel therapeutic targets for immune diseases affecting the central nervous system by focusing on the immune cells (B cells, T cells, monocytes, macrophages, microglia, dendritic cells) and tissue cells (oligodendrocytes, astrocytes, neurons).

This study aimed to investigate transcriptional profile changes of monocytes after exercise using an integrative analysis based on single-cell technology to address the gap in exercise-mediated monocyte prevention and therapy of AD. We also examined the potential expression and regulatory mechanisms of the post-exercise monocyte transcriptome to establish a biological basis for the beneficial mechanism of exercise prescriptions for AD patients.

## Materials and Methods

### Data Acquisition

The dataset was obtained from GEO, an international public open source database (Barrett et al., 2012). The nucleated cell dataset from peripheral blood of three AD patients (aged > 60 years with positive amyloid positron emission tomography) and two age-matched cognitively normal controls (NC) were obtained from GSE181279. The scRNA-seq was used to analyze 36,849 nucleated peripheral blood cells in the GSE181279 dataset through the Cell Ranger standard analysis procedure (Xu and Jia, 2021). The Affymetrix Human Genome U133 Plus 2. 0 Array was used to analyze the GSE51835 dataset, a bulk RNA-seq dataset based on the GPL570 platform. The GSE51835 dataset consisted of mononuclear cells in 24 peripheral blood samples of 12 pre-exercise and 12 post-exercise (20 min) individuals (Radom-Aizik et al., 2014). The humanht-12 v4 Expression bead chip was used to assess GSE140831 dataset, which is based on the GPL15988 platform, using circulating blood mRNA expression data of 204 AD and 530 normal controls. The limma package of the R software was used to analyze variance as previously described. The log fold change (logFC) and mean expression values of all genes were also recorded. Adjusted *p*-values less than 0.05 were considered to be statistically significantly different (Costa-Silva et al., 2017; Chen et al., 2021; Lin W. et al., 2021; Shi et al., 2021; Wu et al., 2021). The “pheatmap” package of the R software was used for heat m*APP*ing. The “rcircos” package was used to draw a circle map describing the chromosomal location of the target gene (Zhang et al., 2013). All raw data were stored in text form.

### Single-Cell Transcriptional Profiling and Clustering

As previously described, scRNA analysis was performed using the Seurat package of R software, which included quality control, downscaling, and clustering of the GSE181279 dataset (Chen et al., 2021; Lin W.-W. et al., 2021; Mangiola et al., 2021; Pereira et al., 2021; Shi et al., 2021; Wu et al., 2021). All genes were expressed in at least three cells. At least 200 genes were expressed in each cell, and the number of mitochondrial genes was less than 10. “FindVariableFeatures” was used to calculate the variable genes between samples after normalization of gene expression. Further non-linear dimensionality reduction analysis was conducted using the UMAP method (Becht et al., 2018). Initial annotation of cell clustering was performed using SingleR (Deng et al., 2020). The cell clusters were further corrected based on the methods described in some previous studies (Zhang et al., 2020; Xu and Jia, 2021). The differential expression analysis of single-cell mimetic bulk RNA data obtained from the specific cells was conducted using DEseq2 to obtain the differentially expressed genes (DEGs) in AD patients (Mou et al., 2020). P-value < 0.05 was considered a statistically significant difference.

### Cell Communication Analysis

Ligand-receptor complex-mediated intercellular communication is key in a wide range of biological processes. In this study, the analysis of intercellular ligand-receptor complex interactions was conducted using the CellPhoneDB database. Intercellular interactions with *p*-values < 0.01 were considered statistically significant (Efremova et al., 2020). Additionally, cell chat was used as an additional method to describe intercellular signaling communication and conserved and context-specific pathways (Jin et al., 2021).

### Scenic-Based Analysis of Key Transcription Factors

Key TFs within single-cell clusters were analyzed using a modified SCENIC method as previously described (20, 22, 33, 34). The regulon activity score (RAS) for each cell was repeated thrice to determine the stability of the regulatory relationship (Suo et al., 2018). An entropy-based strategy was used to quantify the cell-type specificity of the regulation (Cabili et al., 2011). Next, the correlations of the different TF overall regulatory activities were analyzed using Pearson correlation coefficients. It is worth noting that this method can currently only be used to analyze positively associated transcriptional regulatory networks, so only positively associated transcriptional regulators of target genes can be explored.

### Single-Cell Pseudotime Analysis

The pseudotime analysis measures the transcriptional difference a single cell can achieve during cell differentiation. The Monocle package ranks each cell relative to its progress on the learning trajectory. In this study, pseudotime analysis was performed using Monocle2 package (Qiu et al., 2017).

### Enrichment Analysis of Pathways and Functions

Enrichment analyses, including KEGG and gene ontology (GO) were conducted using clusterprofiler package for R software [version 3. 14. 3] (Yu et al., 2012). The org. Hs. eg. db package [version 3. 10. 0] was used for ID conversion. “Homo sapiens” was selected as the species. P-value < 0.05 was considered a statistically significant difference.

### Construction of Predictive Models

The neural network model was constructed as described previously using the R packages “neuralnet” and “neuralnettools” (Beck, 2018). The “pacman” package of the R software was used to construct the random forest (RF) and support vector machine (SVM) models. The models were selected based on the receiver operating characteristic curve (ROC) and the characteristics of the |residuals|. The nomogram prediction model was then built using the “rms” function. Finally, calibration curves for the model were plotted (Kang et al., 2020; Ying et al., 2021). The DCA curves and clinical effectiveness curves were plotted to further evaluate the predictive power of the model (Van Calster et al., 2018; Olivieri, 2021).

## Results

### Transcriptome Analysis of Peripheral Blood Nucleated Cells From Alzheimer’s Disease Patients

**FIGURE 1:**
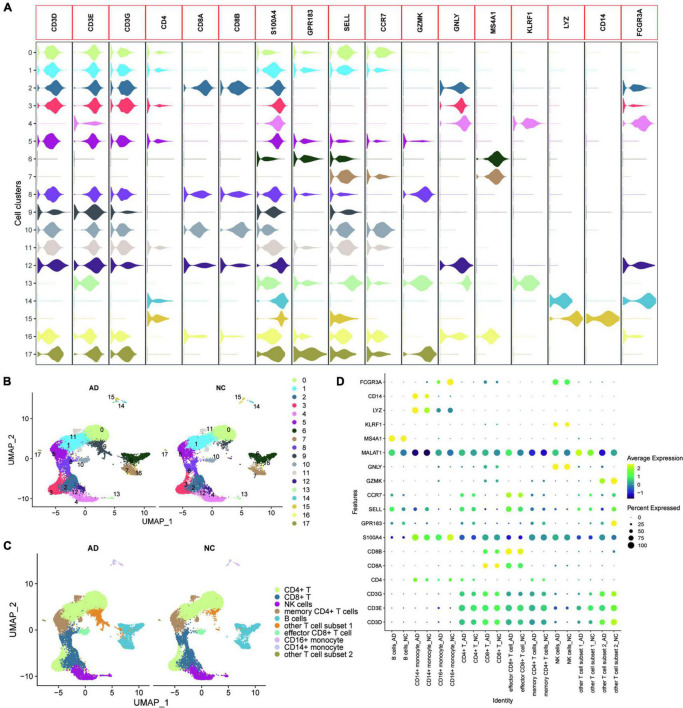
Heterogeneity of the transcriptome of peripheral blood nucleated cells from patients with AD. **(A)** Violin plots showing the expression of the marker genes in different cell clusters; **(B)** UMAPs show the distribution of the 17 cell clusters in the AD and NC groups, with different colors representing different cell clusters; **(C)** The cells are annotated according to the expression characteristics of the cell clusters and marker genes; **(D)** The dotted heat map showing good agreement and characteristic expression of marker genes between AD and NC groups, where MALAT1 as a characteristic gene of other T cell subset 1 was significantly upregulated in other T cell subset 1.

GSE181279 dataset contained 36,849 peripheral blood nucleated cells of three AD patients (aged > 60 years) and two age-matched non-AD patients (NC). All genes were expressed in at least three cells. (Supplementary Figure 1A). At least 200 genes were expressed in each cell, and the number of mitochondrial genes was less than 10. UMAP plots were used to show the distribution of different samples in the tissues of AD and NC groups (Supplementary Figure 1B). Feature RNA, counted RNA, mitochondrial RNA, and hemoglobin RNA are shown in Supplementary Figure 1C. SingleR was used for initial annotation of cell clustering. Key genes defining cell types in circulating blood, including *CD3D*, *CD3E*, *CD3G*, *CD4*, *CD8A*, *CD8B*, *S100A4*, *GPR183*, *SELL*, *CCR7*, *GZMK*, *GNLY*, *MS4A1*, *KLRF1*, *LYZ*, *CD14*, and *FCGR3A* were further determined after initial annotation of cell clustering, based on previous studies (13, 29). Violin plots were used to show the expression of these marker genes in different cell clusters (Figure 1A). The cell types of the different clusters were further annotated based on the expression patterns of the marker genes (Figures 1B,C). The dotted heat map showed that the marker gene expression between the AD and NC groups was consistent, suggesting that this cellular annotation can distinguish different cell types (Figure 1D).

### Cellular Communication Characteristics of CD14^+^ and CD16^+^ Monocytes in Alzheimer’s Disease Based on Exercise

**FIGURE 2:**
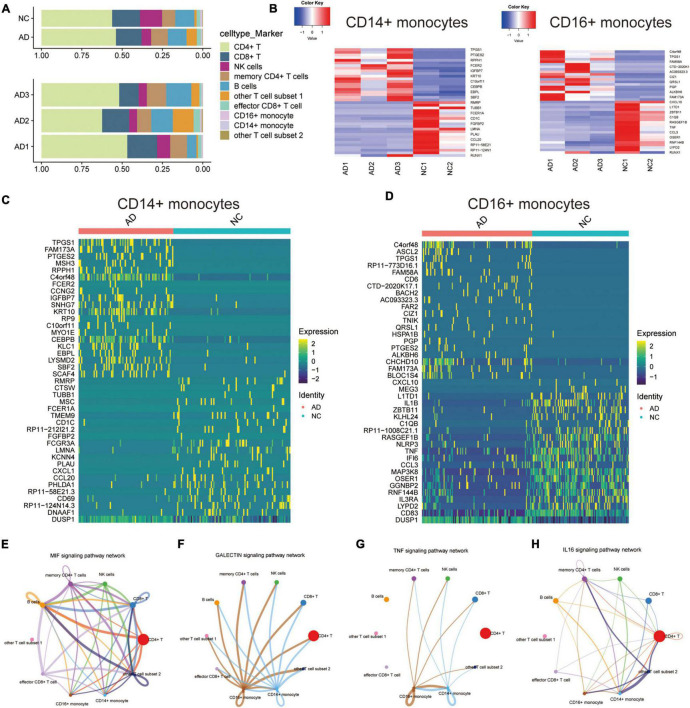
Differential expression and cellular communication characteristics of CD14^+^ and CD16^+^ monocytes in patients with AD. **(A)** Horizontal bars show the proportion of each cell type in different patients; **(B)** Deseq2 obtained the main DEGs in CD14^+^ and CD16^+^ monocytes; **(C,D)** Major DEGs in CD14^+^ and CD16^+^ monocytes obtained by *APP*lying the “FindVariableFeatures” function; **(E–H)** Cellchat-based results showing the association of specific cell populations with other cell populations in different key cellular pathways: MIF signaling pathway **(E)**, GALECTIN signaling pathway **(F)**, *TNF* signaling pathway **(G)**, IL16 signaling pathway **(H)**.

The proportion of each cell type in different patients was expressed in bar chart to analyze the cellular composition changes of circulating blood in AD patients (Figure 2A). The proportions of CD14^+^ and CD16^+^ monocytes were not significantly different between the AD and NC groups. This result indicates that monocyte differences in AD patients are mainly due to transcription differences and not proportion differences of cell types. The “deseq2” package and the “FindVariableFeatures” function were then used to analyze the major differentially expressed genes (DEGs) in CD14^+^ and CD16^+^ monocytes (Figures 2B–D). Cellchat was used to analyze the communication between CD14 + and CD16 + monocytes and other cells along a number of pathways, including the MIF signaling pathway (Figure 2E), GALECTIN signaling pathway (Figure 2F), *TNF* signaling pathway (Figure 2G), IL16 signaling pathway (Figure 2H), ANNEXIN signaling pathway (Supplementary Figure 2A), RESISTIN signaling pathway was assessed using Cellchat (Supplementary Figure 2B). This suggests that CD14 + and CD16 + monocytes are communicate with other cells, and that monocytes may regulate the circulating blood microenvironment.

**FIGURE 3:**
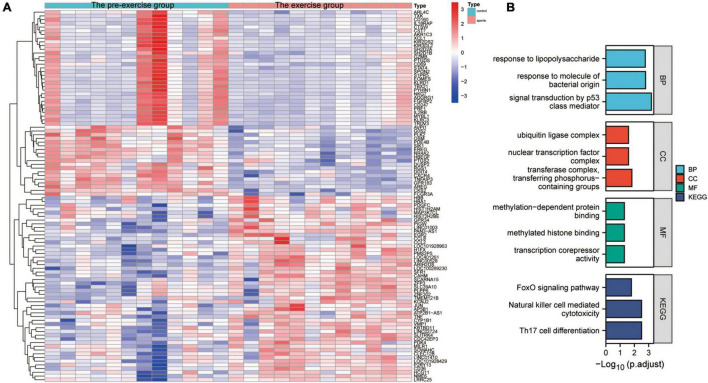
Characterization of the effect of the exercise group on the expression profile of monocytes. **(A)** Heat map of the major DEGs in monocytes from the GSE51835 bulk RNA dataset before versus after the patients received exercise; **(B)** GO and KEGG enrichment analysis of the major DEGs in monocytes.

The effect of exercise on monocyte transcription in circulating blood was evaluated to analyze the mechanisms by which exercise regulates mRNA transcription levels in monocytes of AD patients. The “limma” package was used for differential analysis of the GSE51835 bulk RNA-seq, and 1811 DEGs were obtained. The main DEGs are shown on the heat map (Figure 3A). GO, and KEGG enrichment analyses of these DEGs are shown in Figure 3B. The GO functional enrichment analysis showed that the DEGs were significantly enriched in: response to lipopolysaccharide, response to molecule of bacterial origin, signal transduction by p53 class mediator, ubiquitin ligase complex, nuclear transcription factor complex, transferase complex, transferring phosphorus-containing groups, methylation-dependent protein binding, methylated histone binding, and transcription corepressor activity. Moreover, the KEGG enrichment analysis showed that the DEGs were significantly enriched in the FOXO signaling pathway, Natural killer cell-mediated cytotoxicity, and Th17 cell differentiation. The enrichment analyses suggest that transcriptional and immune microenvironmental homeostasis regulation are the main effects of exercise on monocytes (Figure 3B). A total of 57 and 39 DEGs were associated with AD at the single-cell level in CD14^+^ and CD16^+^ monocytes, respectively (Supplementary Figure 2C). GO, and KEGG enrichment analyses showed that these DEGs were mainly enriched in functions and pathways related to immune differentiation and transcriptional regulation (Supplementary Figures 2D,E). These analyses indicate that CD14^+^ and CD16^+^ monocytes play a crucial cellular communication function in regulating blood microenvironment in AD. These results show that exercise can influence the immune microenvironment in circulating blood by regulating the transcription of monocytes, and thus may be a mechanism for AD prevention through exercise. However, further studies are needed to assess the effect of exercise on monocyte transcriptional regulators to provide an initiating factor for the prevention of AD development through exercise. Moreover, further exploration of exercise-regulated changes in cellular communication can reveal the specific mechanisms by which these transcriptional regulators modulate the blood microenvironment.

### *TNF, CCR1, APP*, and *AREG* Are Key Ligand-Receptor-Related Genes Associated With Exercise Prescription

**FIGURE 4:**
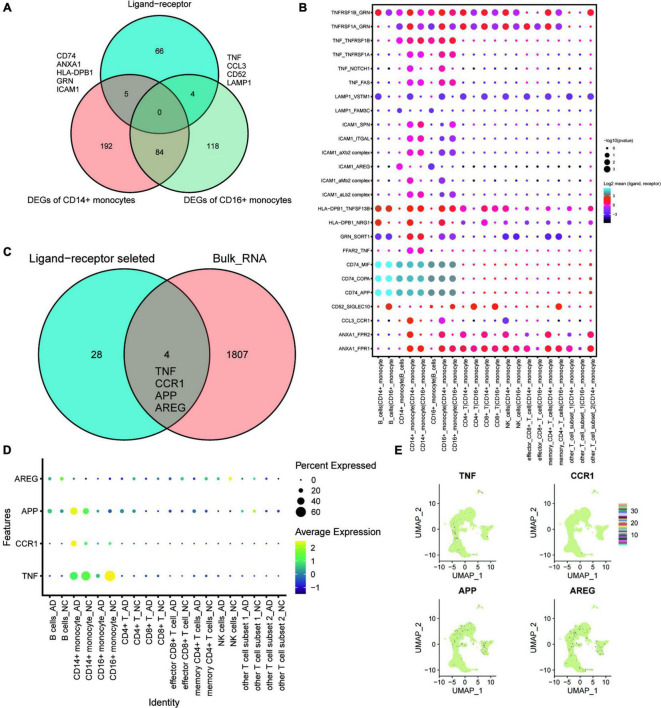
The key ligand-receptor-related genes associated with exercise prescription. **(A)** Venn diagram analysis identifies CD74, ANXA1, HLA-DPB1, GRN, and ICAM1 as the intersections of DEGs of CD14^+^ monocytes with intersecting genes of key ligand-receptors, whereas the *TNF*, CCL3, CD52, and LAMP1 were intersections of DEGs of CD16^+^ monocytes with key ligand-receptor intersection genes; **(B)** Pointwise heat map of key ligand-receptor relationship pairs associated with CD74, ANXA1, HLA-DPB1, GRN, ICAM1, *TNF*, CCL3, CD52, and LAMP1. **(C)** Wayne plots showing that *TNF*, *CCR1*, *APP*, and *AREG* in these selected ligand-receptor relationship pairs were found to be differentially expressed in post-exercise monocytes; **(D)** Dot heat map demonstrating the differential expression profile of *TNF*, *CCR1*, *APP*, and *AREG* genes in the AD and NC groups; **(E)** Transcriptional features of *TNF*, *CCR1*, *APP*, and *AREG* genes in the GSE181279 single cell dataset demonstrated using UMAP plots.

Ligand-receptor complex-mediated intercellular communication is essential for various biological processes. This study conducted intercellular ligand-receptor complex interaction analysis using the CellPhoneDB database. Any pair with *p* < 0.05 was considered key ligand-receptor pair. Intersection analysis was performed to explore cellular communication changes under exercise regulation (shown in the Venn diagram, Figure 4A). *CD74*, *ANXA1*, *HLA-DPB1*, *GRN*, and *ICAM1* were DEGs of CD14^+^ monocytes intersecting with key ligand-receptor intersection genes. Moreover, *TNF*, *CCL3*, *CD52*, and *LAMP1* were DEGs of CD16^+^ monocytes intersecting with key ligand-receptor intersection genes. A dotted heat map of key ligand-receptor relationship pairs associated with *CD74*, *ANXA1*, *HLA-DPB1*, *GRN*, *ICAM1*, *TNF*, *CCL3*, *CD52*, and *LAMP* is shown in Figure 4B. Of the 32 ligand-receptor relationship pairs, exercise regulated the transcript levels of four genes (*TNF*, *CCR1*, *APP*, and *AREG*). A dotted heat map demonstrated the differential expression profile of *TNF*, *CCR1*, *APP*, and *AREG* genes in the AD and normal control (NC) groups (Figure 4D). UMAP plots were used to demonstrate the transcriptional profiles of *TNF*, *CCR1*, *APP*, and *AREG* in the GSE181279 single-cell dataset (Figure 4E), suggesting that *TNF*, *CCR1*, *APP*, and *AREG* may be key ligand-receptor-related genes associated with exercise prescription.

### *MAFB*, *HES4*, and *FOSL1* Are Differentially Expressed Transcription Factors in Alzheimer’s Disease

**FIGURE 5:**
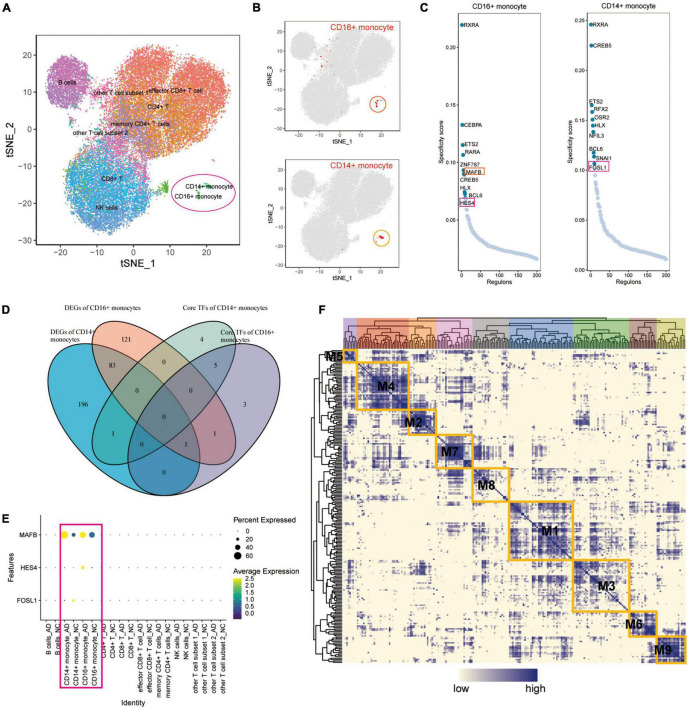
The key transcription factors of monocytes affected by exercise in patients with AD. **(A)** Unsupervised clustering analysis of the GSE181279 dataset was re-performed using the t-SNE method; **(B)** The t-SNE plot showing the clustering results of CD14^+^ and CD16^+^ monocytes, which suggest that the monocytes are well clustered; **(C)** The top 10 TFs of CD14^+^ and CD16^+^ monocytes based on RSS ranking positions; **(D)** Venn diagram of intersection analysis of DEGs in CD14^+^ and CD16^+^ monocytes from patients with AD and having key TFs in top10 of CD14^+^ and CD16^+^ monocytes; **(E)** Dotted heat map demonstrating the differential expression profile of *MAFB*, *HES4*, and *FOSL1* genes in the AD and NC groups; **(F)** Expression of transcription factors quantified using an entropic strategy and re-clustered in the same way.

A SCENIC analysis was performed on all cells of GSE181279 to further identify differentially expressed transcription factors in AD associated with exercise. An unsupervised cluster analysis of the GSE181279 dataset was re-performed using the t-SNE method. The t-SNE plot demonstrated the clustering results for CD14^+^ and CD16^+^ monocytes (Figures 5A,B), suggesting that CD14^+^ and CD16^+^ monocytes were well clustered. According to SCENIC analysis, the top 10 TFs of CD14^+^ and CD16^+^ monocytes were ranked based on the regulon specificity score (RSS; Figure 5C). Venn diagrams of DEGs in CD14^+^ and CD16^+^ monocytes from AD patients were plotted against the top 10 key TFs in CD14^+^ and CD16^+^ monocytes via intersection analysis to screen the key transcriptional regulators that are differentially expressed in the AD blood microenvironment (Figure 5D). These 10 key transcription factors could be possible targets for exercise to regulate AD onset if exercise can influence AD onset by modulating transcription factors in monocytes. *MAFB*, *HES4*, and *FOSL1* were differentially expressed in AD. The dotted heat map demonstrated the differential expression profile of *MAFB*, *HES4*, and *FOSL1* genes in the AD and NC groups (Figure 5E), indicating that these genes are transcription factors differentially expressed in AD patients.

### Transcription Factors in M4 Cluster Are Target Genes for Exercise Regulation of Alzheimer’s Disease Monocytes

**FIGURE 6:**
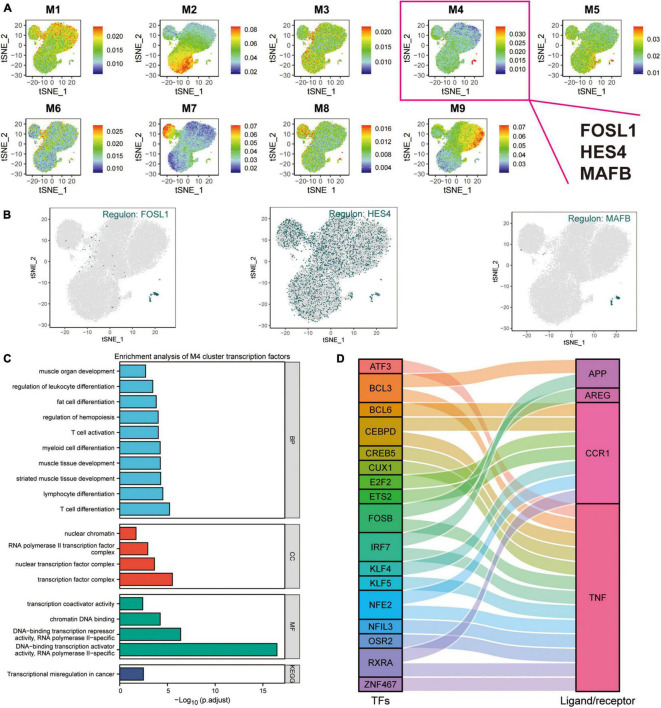
Cluster analysis of target genes in exercise-regulated AD monocytes. **(A)** The transcriptional activity profile of the M1–M9 module is illustrated using t-SNE plots, where *FOSL1*, *HES4*, and *MAFB* are the signature transcription factors of the M4 module; **(B)** Expression profiles of key transcription factors *FOSL1*, *HES4*, and *MAFB* are shown using t-SNE plots; **(C)** GO and KEGG enrichment analysis of M4 transcription factor clusters; **(D)** Sangi diagram demonstrating the key ligands or receptors that may be regulated by the transcription factors in the M4 cluster.

Transcription factors represent functional and differentiation trajectories of cells. Herein, the expression of transcription factors was quantified using an entropic strategy, then underwent re-cluster analysis to determine the transcription factor clusters where the transcriptional regulators *MAFB*, *HES4*, and *FOSL1* are located (Figure 5F). We used the UMAP method for the cell type clustering analysis, and the tSNE clustering strategy for the transcriptional regulatory network analysis. The t-SNE plots were used to characterize the transcriptional activity of the M1–M9 modules, where *FOSL1*, *HES4*, and *MAFB* were found to be the characteristic transcription factors of the M4 module (Figures 6A,B and Supplementary Table 2). In this investigation, both UMAP and tSNE clustering methods performed well, examined the effect of hierarchical clustering in this study. The transcriptional activity of the various cell types in the different cell modules is shown in Figures 6A,B. CD14^+^ and CD16^+^ monocytes were significantly enriched in the M4 module (Supplementary Figure 3A). GO, and KEGG enrichment analyses were used to explore the function of transcription factors in the M4 cluster (Figure 6C). These transcription factors were found to play a role in muscle organ development, regulation of leukocyte differentiation, fat cell differentiation, hemopoiesis regulation, T cell activation, myeloid cell differentiation, muscle tissue development, lymphocyte differentiation, T cell differentiation, nuclear chromatin, RNA polymerase Il transcription factor complex, nuclear transcription factor complex, transcription factor complex, transcription coactivator activity, and chromatin DNA binding. Moreover, the M4 cluster was significantly enriched in various pathways and functions, such as chromatin DNA binding, and DNA-binding transcription repressor activity. These results suggest that transcription factors in the M4 cluster are closely related to muscle tissue development, immune cell differentiation, and cellular transcriptional regulatory functions and pathways. *TNF*, *CCR1*, *APP*, and *AREG* were identified as key ligand-receptor-related genes associated with exercise prescription. Sankey maps were drawn based on the results of previous SCENIC analyses to reveal transcriptional regulators that may regulate *TNF*, *CCR1*, *APP*, and *AREG* (Figure 6D). Differential analysis by bulkRNA-seq revealed that the expression of TNF, CCR1, and APP were all upregulated after exercise (*p* < 0.05). These findings show that transcription factors in the M4 cluster (TNF, CCR1, and APP) may have a role in the prevention of Alzheimer’s disease through exercise.

### Construction of a Transcriptional Regulatory Network for Exercise-Altered Alzheimer’s Disease Monocytes via Combined Bulk RNA-seq and Single-Cell Mimetic Timing Analysis

**FIGURE 7:**
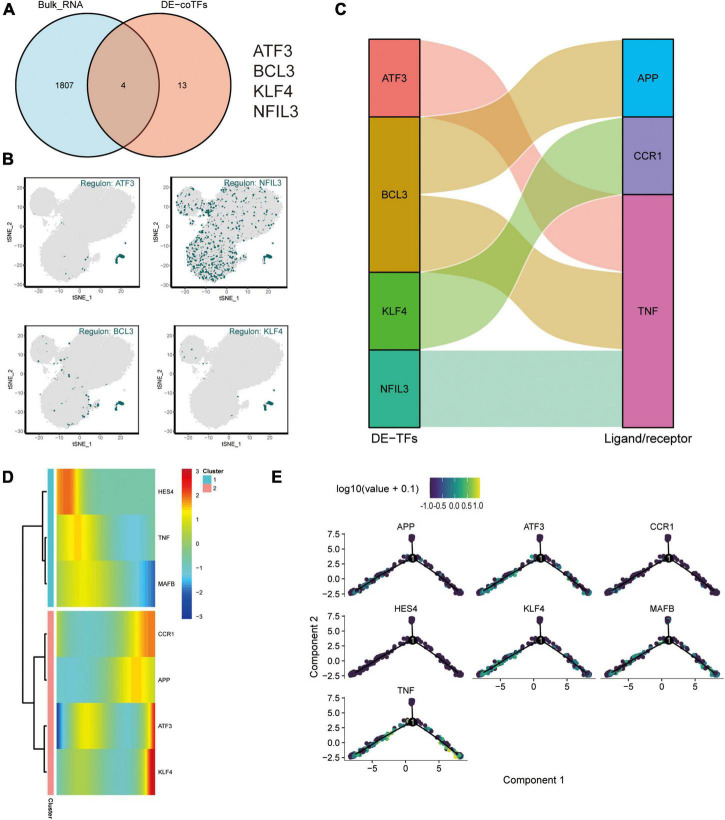
Exercise regulates AD monocyte transcription (ERADMT) gene sets. **(A)** Intersection analysis of 17 key transcription factors from the M4 cluster with 1811 DEGs after exercise; **(B)** Expression characteristics of the intersecting genes *ATF3*, *NFIL3*, *BCL3*, and *KLF4* on the t-SNE plot; **(C)** Sangi diagram showing the key ligands or receptors (including: *APP*, *CCR1*, and *TNF*) that may be regulated by the co-differentially expressed transcription factors (including: *ATF3*, *BCL3*, *KLF4*, and *NFIL3*); **(D)** Heat map indicating the relationship between *APP*, *CCR1*, *TNF*, *ATF3*, *KLF4*, *HES4*, and *MAFB* expression and pseudo-time; **(E)** Expression characteristics of *APP*, *CCR1*, *TNF*, *ATF3*, *KLF4*, *HES4*, and *MAFB* on the pseudotime trajectory.

And an intersection analysis of 17 key transcription factors from the M4 cluster with 1811 DEG that were differentially expressed after exercise was performed to screen the key differentially expressed transcriptional regulators in the AD blood microenvironment affected by exercise (Figure 7A). The transcription factors *ATF3*, *BCL3*, *KLF4*, and *NFIL3* were the key TFs regulated by AD and exercise. The expression profiles of *ATF3*, *NFIL3*, *BCL3*, and *KLF4* on the t-SNE plot are shown in Figure 7B. The key transcription factors were predominantly expressed in CD14^+^ and CD16^+^ monocytes. Subsequently, the sankey diagram demonstrated the key ligands or receptors (*APP*, *CCR1*, and *TNF*) that may be regulated by the co-differentially expressed transcription factors (*ATF3*, *BCL3*, *KLF4*, and *NFIL3*; Figure 7C). *APP*, *CCR1*, *TNF*, *ATF3*, *KLF4*, *HES4*, and *MAFB* were considered exercise-regulated AD monocyte transcription (ERADMT) gene sets. Pseudo-temporal analysis measures the transcriptional variation that can be achieved by single cells during cell differentiation. In this study, pseudo-timing analysis was conducted using the Monocle2 package. First, a principal component (PCA) analysis of all CD14^+^ and CD16^+^ monocytes was performed (Supplementary Figure 3B), and results showed that CD14^+^ and CD16^+^ monocytes could be distinguished. Scatter plots were used to further characterize the expression profiles of *APP*, *CCR1*, *TNF*, *ATF3*, *KLF4*, *HES4*, and *MAFB* (Supplementary Figure 3C). The distribution of CD14^+^, CD16^+^ monocytes, and pseudotime values and the branching of the proposed chronological trajectory were also further plotted on the proposed chronological trajectory (Supplementary Figure 3D). The distribution of *APP*, *CCR1*, *TNF*, *ATF3*, *KLF4*, *HES4*, and *MAFB* expression values on the pseudo-temporal trajectory based on pseudo-time was also assessed (Figures 7D,E) (Supplementary Figure 3E). The ERADMT gene set of *APP*, *CCR1*, *TNF*, *ATF3*, *HES4*, and *KLF4* were significantly differentially expressed in the post-exercise bulk RNA dataset (Supplementary Figure 3F). Furthermore, ERADMT gene set was concentrated in the state 1 branch of the pseudo-temporal track and enriched in CD14^+^ monocyte. These results indicate that exercise can improve the microenvironment in AD circulating blood mainly by driving CD14^+^ monocyte.

### ERADMT Gene Set Can Be Used as an Indicator for Assessing Exercise Therapy Compliance in Alzheimer’s Disease Patients

**FIGURE 8:**
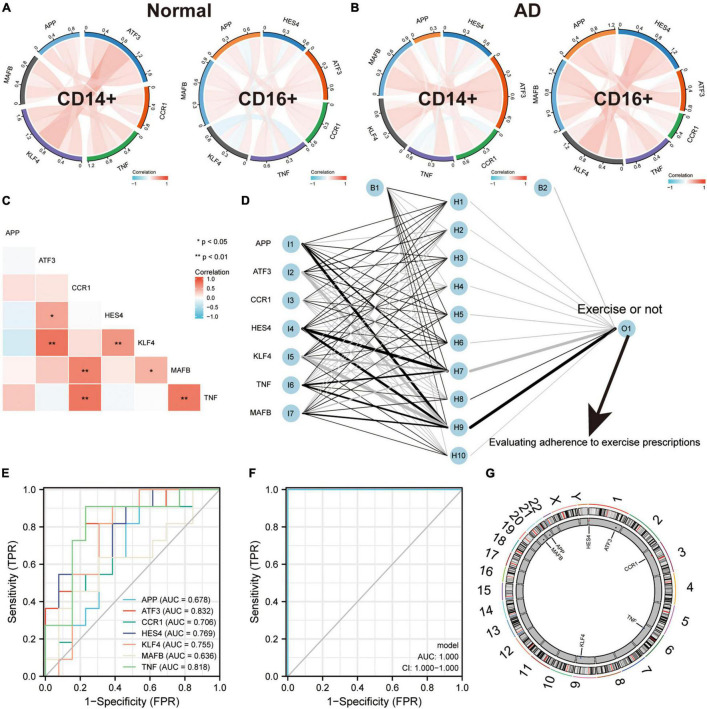
ERADMT gene set as an indicator for assessing adherence to exercise prescription in patients with AD. **(A,B)** Correlation of *APP*, *CCR1*, *TNF*, *ATF3*, *KLF4*, *HES4*, and *MAFB* expression in CD14^+^ and CD16^+^ monocytes based on the GSE181279 single cell dataset, analyzed using Person method, including NC group (A) and AD group (B), red indicates positive correlation whereas blue indicates negative correlation; **(C)** Expression correlations of *APP*, *CCR1*, *TNF*, *ATF3*, *KLF4*, *HES4*, and *MAFB* in monocytes were analyzed using the Person method based on the GSE181279 bulk RNA dataset; **(D)** Schematic diagram of a neural network model for determining whether to exercise using *APP*, *CCR1*, *TNF*, *ATF3*, *KLF4*, *HES4*, and *MAFB*; **(E)** The predictive ability of the ERADMT gene set (*APP*, *CCR1*, *TNF*, *ATF3*, *KLF4*, *HES4*, and *MAFB*) to separately predict whether to undergo exercise or not; **(F)** ROC curve indicating that the neural network model can accurately predict patients’ exercise compliance; **(G)** The position of the ERADMT gene set (*APP*, *CCR1*, *TNF*, *ATF3*, *KLF4*, *HES4*, and *MAFB*) on the chromosome is indicated by the circle plot.

**FIGURE 9:**
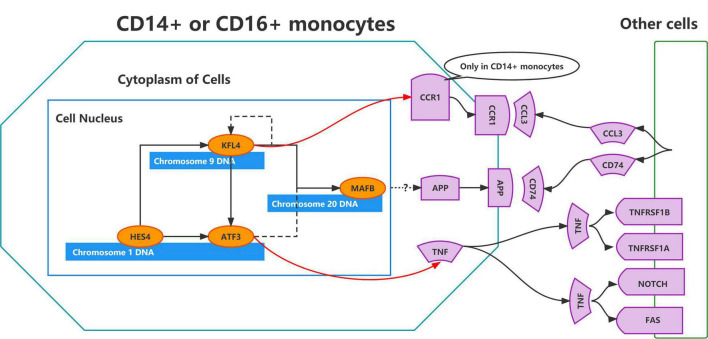
Schematic representation of the regulatory network constructed using the ERADMT gene set.

To validate the regulatory relationships in the ERADMT gene set, we analyzed the expression correlation of *APP, CCR1, TNF, ATF3, KLF4, HES4*, and *MAFB* in CD14 + and CD16 + monocytes using the Pearson correlation method based on the GSE181279 single cell dataset (Figures 8A,B). In addition, we also analyzed the correlations among the expression levels of *APP, CCR1, TNF, ATF3, KLF4, HES4*, and *MAFB* in monocytes based on the GSE51835 bulk RNA dataset using the Pearson correlation method (Figure 8C). The results were similar to those of the SCENIC analysis, i.e., The expression of genes in the ERADMT gene set was positively correlated with each other’s expression. To assess the function of these monocyte genes for exercise adherence, a neural network model was constructed using *APP, CCR1, TNF, ATF3, KLF4, HES4*, and *MAFB* (Figure 8D). In addition, ROC analysis suggested that all elements in the ERADMT gene set had good ability to assess whether exercise was received (Figure 8E). The ROC curves indicated that the neural network model could accurately assess patients’ exercise compliance (Figure 8F). The location of the ERADMT gene set (*APP, CCR1, TNF, ATF3, KLF4, HES4*, and *MAFB*) on the chromosomes in this study is also shown as a circle plot (Figure 8G). The above study suggests that the level of expression of genes in the ERADMT gene set may reflect exercise therapy adherence in AD patients. It can also predict whether the subjects are receiving adequate amounts of physical activity. In summary, *ATF3*, *MAFB*, *HES4*, and *KLF4* were defined as potential transcription factors for *TNF*, *CCR1* and *APP* under the regulation of exercise prescription by Bulk RNA-seq and single cell mimetic timing analysis. Accordingly, a network of exercise-altered monocyte transcriptional regulation was constructed (Figure 9).

### ERADMT Gene Set Found to Be a Potential Risk Marker for the Development of Alzheimer’s Disease Patients

To further assess the predictive power of the ERADMT gene set for the onset of AD in patients, we performed further analysis with the GSE140831 dataset. We obtained circulating blood mRNA expression data from a total of 204 patients with Alzheimer’s disease and 530 normal controls. Correlation analysis based on the GSE140831 dataset suggested that the expression in the ERADMT gene set was positively correlated with each other, except for *APP*, which was negatively correlated with *ATF3* and *HES4* (Supplementary Figure 4A). Next, RF models and SVM models were constructed to assess the predictive power of the ERADMT gene set on the onset of AD patients. The box plot and Reverse cumulative distribution of |residual| indicate that the model residuals of the SVM model are larger than those of the RF model (Supplementary Figures 4B,C). The relationship between the “error” of the RF model and the number of trees selected for the model is shown in Supplementary Figure 4D. The importance of ERADMT genes was also ranked (Supplementary Figure 4E). The AUC values of the RF model were significantly higher than those of the SVM model (Supplementary Figure 4F). To visualize the model, we constructed a nomogram prediction model using the ERADMT gene set expression profile (Supplementary Figure 4G). The calibration curve of this ERADMT gene set nomogram prediction model suggests that the model prediction results fit well with the actual results (Supplementary Figure 4H). The DCA curve of the nomogram prediction model based on this ERADMT gene set suggests that the model has potentially good clinical utility (Supplementary Figures 5A,B). According to the results of the logistic prediction model, the ERADMT gene set is a potential risk marker for the development of AD patients.

## Discussion

Peripheral blood nucleated cells of AD patients annotated nine cell subsets, including CD4^+^ T cells, CD8^+^ T cells, NK cells, memory CD4^+^ T cells, B cells, effector CD8^+^ T cells, CD14^+^ monocytes, CD16^+^ monocytes, other T cell subset 1 and other T Cell subset 2. Cell communication analysis revealed that CD14^+^ and CD16^+^ monocytes have a close cellular communication with other cells. CD14^+^ and CD16^+^ monocytes play a key cellular communication function in the regulation of the AD blood microenvironment, consistent with previous studies (Gu et al., 2016; Kapellos et al., 2019). Moreover, four genes (*TNF*, *CCR1*, *APP*, and *AREG*) were identified as potentially key ligand-receptor-related genes associated with exercise prescription. Enrichment analyses suggested that regulation of transcriptional and immune microenvironment homeostasis are the main effects of exercise on monocytes, indicating the potential mechanism for AD prevention. SCENIC analysis identified *MAFB*, *HES4*, and *FOSL1* as differentially expressed transcription factors in AD patients. Moreover, *FOSL1*, *HES4*, and *MAFB* were identified as characteristic transcription factors of the M4 module. The transcription factors in the M4 cluster were closely associated with muscle tissue development, immune cell differentiation, and transcriptional regulatory functions and pathways of cells. These results suggest that the transcription factors in the M4 cluster may be key target genes for exercise regulation.

The expression of transcription factors in each module had a substantial positive connection, and *FOSL1*, *HES4*, and *MAFB* identified the genes in the M4 module as a possible cluster of transcriptional regulators linked to the start of Alzheimer’s disease. M4 module genes *ATF3*, *BCL3*, *KLF4*, and *NFIL3* are differentially expressed in the exercise group; therefore, *ATF3*, *BCL3*, *KLF4*, and *NFIL3* are regarded pivotal genes in the prevention of AD incidence by exercise. Therefore, *APP*, *CCR1*, *TNF*, *ATF3*, *KLF4*, *HES4*, and *MAFB* were identified as ERADMT gene set. ERADMT gene set, as a set of genes in the regulatory network, is a potential risk marker for AD development and an indicator for the assessment of exercise therapy adherence in AD patients.

The pseudo-timing analysis showed that ERADMT gene set was concentrated in the state 1 branch of the pseudo-timing trajectory and enriched in CD14^+^ monocyte, indicating that exercise can improve the microenvironment in AD circulating blood mainly by driving CD14^+^ monocyte. The ERADMT gene set was also concentrated in the state 1 branch of the pseudo-temporal trajectory and enriched in CD14^+^ monocyte, indicating that exercise can improve the microenvironment in AD circulating blood mainly by driving CD14^+^ monocyte. Neural network models were then constructed using *APP*, *CCR1*, *TNF*, *ATF3*, *KLF4*, *HES4*, and *MAFB*. Subsequently, *ATF3*, *MAFB*, *HES4*, and *KLF4* were identified as potential transcription factors for *TNF*, *CCR1* and *APP* under the regulation of exercise prescription. A network of exercise-altered transcriptional regulation of monocytes was also constructed. Finally, predictive models constructed based on RF and SVM suggested that the ERADMT gene set is a potential risk marker for AD development.

The three TFs (*ATF3*, *KFL4*, and *HES4*) were located in the M4 cluster and enriched in AD-related pathways. A previous study showed that *ATF3* is a TF regulated by locomotion (Park et al., 2021). Moreover, exercise upregulates *ATF3* transcription in skeletal muscle after exercise (Fernández-Verdejo et al., 2017). Treadmill exercise can promote sciatic nerve injury regeneration through *ATF3* upregulation (Kim et al., 2020). In contrast, silencing of the *ATF3* family gene can inhibit exercise-induced angiogenesis (Fan et al., 2021). *ATF3* can also promote the progression of neurodegenerative diseases (Bao et al., 2021). Previous studies have revealed that KFL4, as a tumor stem cell marker, promotes cell differentiation and development (Li et al., 2019; Chen et al., 2020; Paterson et al., 2021). It modulates the development of non-hematopoietic cell lineage and is also regarded as a cellular marker (Pessina et al., 2010). However, in a recent study, KFL4 was found to be associated with the onset of dementia, which is consistent with our findings (Santiago et al., 2020). This is the first study to suggest that motor regulation can affect *KFL4*. *HES4* is a key transcription factor in the NOTCH pathway (Wagley et al., 2020) that promotes the initiation of early t-cell development. It may also be associated with mediation of NOTCH pathway-dependent differences in human hematopoietic cell lines (De Decker et al., 2020). Epigenetic dysregulation of *HES4* may be closely associated with the development of Huntington’s disease in the nervous system (Bai et al., 2015). In the present study, *ATF3*, *KFL4*, and *HES4* were identified for the first time as potential TFs mediating the effects of exercise therapy.

Moreover, *APP*, *CCR1*, and *TNF* were identified as key ligand-receptor genes. *APP* was differentially expressed after exercise. *APP* mutations cause AD (Lee et al., 2018). Amyloid hypothesis shows that secretases cleave APP to form toxic amyloid-B (AB) peptides and plaques, thus causing AD (Selkoe and Hardy, 2016). Herein, *APP* expression and its expression ratio in CD14^+^ monocytes were significantly increased in AD patients. The monocyte-macrophage-microglial cell lineage of the cerebrovascular pericyte lineage produces fibrillar A beta in the capillary wall (Wisniewski et al., 2000). *APP* is significantly upregulated in CD14^+^ monocytes of AD patients, indicating that *APP* may be a key cause of amyloid deposition. Moreover, this study showed that the key transcription factor ATF3 can positively regulate the key ligand TNF. TNF is a ligand for TNFRSF1B, TNFRSF1A, NOTCH and FAS. In a previous study on the GSE51835 dataset, it was found that high-intensity exercise may influence the monocyte immune function by regulating ATF3 via the TNF and FoxO signaling pathways. Hence, it may improve the body’s resistance to chronic inflammatory diseases (Li and Luo, 2021). Chemokine and its receptors play a key role in the development of neurodegenerative diseases (Savarin-Vuaillat and Ransohoff, 2007). CCR1 is a receptor for CCL3. Herein, KLF4 could positively regulate the key receptor CCR1. It was earlier reported that CCR1 was a potential target in AD. However, the relationship between CCR1 and exercise has not been clarified (Liu et al., 2014). Overall, the key ligand-receptor genes (*APP*, *CCR1*, and *TNF*) play crucial cellular communication functions in the regulation of the AD blood microenvironment. Exercise can affect the immune microenvironment in circulating blood by regulating the transcription of monocytes, and thus may be a potential mechanism for AD prevention through exercise.

In this study, the integration analysis of scRNA-seq and bulk RNA-seq data revealed that exercise improves cellular communication in circulating blood of AD patients probably by altering the transcriptional regulatory network of monocytes. Moreover, the M4 clustered TFs were enriched in AD-related pathways. In the M4 cluster, ATF3, MAFB, HES4, and KLF4 TFs regulated the transcription of exercise-related ligand receptor genes (*TNF, CCR1*, and *APP*). The ERADMT gene set comp’rises *APP, CCR1, TNF, ATF3, KLF4, HES4*, and *MAFB.* Abnormal function of APP protein has been linked to extracellular β-amyloid deposition. Further analysis revealed that the ERADMT gene set is a potential indicator of exercise therapy adherence in AD patients and a risk marker for AD development. In conclusion, the proposed ERADMT gene set regulated by locomotion provides insights for further mechanistic studies. Although this study included three datasets with large sample sizes and multidimensional and multilevel analyses were performed, this study, as a bioinformatic analysis, requires further experimental validation. Additionally, further clinical studies are needed.

## Conclusion

This study constructed a network of movement-altered AD monocyte transcriptional regulatory features using single-cell integration analysis, mainly composed of ERADMT gene set (*APP*, *CCR1*, *TNF*, *ATF3*, *KLF4*, *HES4*, and *MAFB*). The results showed that the ERADMT gene set can be potential markers for AD development and an indicator of adherence to exercise therapy in AD patients.

## Data Availability Statement

The datasets presented in this study can be found in online repositories. The names of the repository/repositories and accession number(s) can be found in the article/Supplementary Material.

## Author Contributions

YC: methodology, writing – review and editing, conceptualization, software, validation, formal analysis, data curation, and writing – original draft. YS: conceptualization, methodology, supervision, and writing – original draft. ZL: methodology, conceptualization, and writing – original draft. XC: supervision, writing – review and editing. YW: supervision, writing – review and editing. BQ: data curation, writing – review and editing. JL: data curation and methodology. W-WL: software, validation, and language polishing. CS: validation and language polishing. YZ: conceptualization and validation. JH: validation and data curation. YX: writing – original draft, conceptualization, supervision, methodology, and funding acquisition. JC: conceptualization, supervision, project administration, and funding acquisition. SC: conceptualization, supervision, project administration and funding acquisition.

## Conflict of Interest

The authors declare that the research was conducted in the absence of any commercial or financial relationships that could be construed as a potential conflict of interest.

## Publisher’s Note

All claims expressed in this article are solely those of the authors and do not necessarily represent those of their affiliated organizations, or those of the publisher, the editors and the reviewers. Any product that may be evaluated in this article, or claim that may be made by its manufacturer, is not guaranteed or endorsed by the publisher.

## Supplementary Material

Supplementary Figure 1 Quality control for the scRNA-seq data of GSE181279 dataset. **(A)** The plot on the left represents a scatter plot of the number of mitochondria in individual cells and number of RNAs detected. The plot in the middle represents a scatter plot of the number of signature genes in relation to the number of RNAs detected. The plot on the right represents a scatter plot of the number of erythrocyte proteins mixed in the sample in relation to number of RNAs detected; **(B)** Distribution of all clusters is represented with a UMAP plot, where different colors represent different samples; **(C)** Differences in signature RNA expression, the total number of RNAs detected, pMT, and pHB in different clinical samples. pMT: percent of mitochondrial counts; pHB: percent of hemoglobin RNA counts.

Supplementary Figure 2 Intersection and enrichment analysis of DEGs in CD14^+^ and CD16^+^ monocytes from Alzheimer’s Disease (AD) patients for the GSE51835 bulk RNA dataset. **(A,B)** Cellchat-based results demonstrating the association of specific cell populations with other cell populations on various cellular pathways, including the ANNEXIN signaling pathway (A), and RESISTIN signaling pathway (B); **(C)** Venn diagram showing results of intersection analysis of DEGs in CD14^+^ and CD16^+^ monocytes from AD patients for the GSE51835 bulk RNA dataset; **(D)** Differentially expressed genes co-expressed in CD14 + monocytes from AD patients after exercise; **(E)** Differentially expressed genes co-expressed in CD16 + monocytes from AD patients after exercise.

Supplementary Figure 3 Key transcriptome clusters of monocytes affected by exercise in AD patients with pseudotime analysis. **(A)** Distinct cell types’ transcriptional regulatory activity in different cell modules; **(B)** All CD14^+^ and CD16^+^ monocytes were subjected to a principal component analysis (PCA); **(C)** Scatter plots of *APP*, *CCR1*, *TNF*, *ATF3*, *KLF4*, *HES4*, and *MAFB* expression profiles; **(D)** Distribution of CD14^+^ and CD16^+^ monocytes on pseudotime trajectories, pseudotime value distribution plots, and pseudotime trajectories branching **(E)**. *APP*, *CCR1*, *TNF*, *ATF3*, *KLF4*, *HES4*, and *MAFB* expression levels in relation to pseudo-time; **(F)** In the post-exercise bulk RNA collection, these seven important genes of *APP*, *CCR1*, *TNF*, *ATF3*, *HES4*, and *KLF4* were discovered to be tightly related.

Supplementary Figure 4 The ERADMT gene set was found to be a potential risk marker for the development of patients with AD. **(A)** Correlation analysis of *APP*, *CCR1*, *TNF*, *ATF3*, *KLF4*, *HES4*, and *MAFB* in the GSE140831 dataset; **(B,C)** The box plot shows that the model residuals of SVM are larger than those of RF model; **(D)** Relationship between the “error” of the RF model and number of selected trees of the model; **(E)** Ranking of ERADMT genes according to their importance; **(F)** Predictive power of RF and SVM models with predictive power demonstrated by the ROC curves; **(G)** The nomogram prediction model constructed with the expression features of the ERADMT gene set; **(H)** The nomogram prediction model’s calibration curve based on the ERADMT gene set.

Supplementary Figure 5 The ERADMT gene set prediction AD risk column nomogram prediction model’s predictive power was evaluated; **(A)** Decision Curve Analysis (DCA) curves of the nomogram prediction model based on the ERADMT gene set; **(B)** The nomogram prediction model based on the ERADMT gene set underwent DCA.
