# Supplementary figures and images for: Exercise Modifies the Transcriptional Regulatory Features of Monocytes in Alzheimer’s Patients: A Multi-Omics Integration Analysis Based on Single Cell Technology
Source: Front Aging Neurosci. 2022 May 3;14:881488. doi: 10.3389/fnagi.2022.881488 (PMC9110789; doi:10.3389/fnagi.2022.881488)

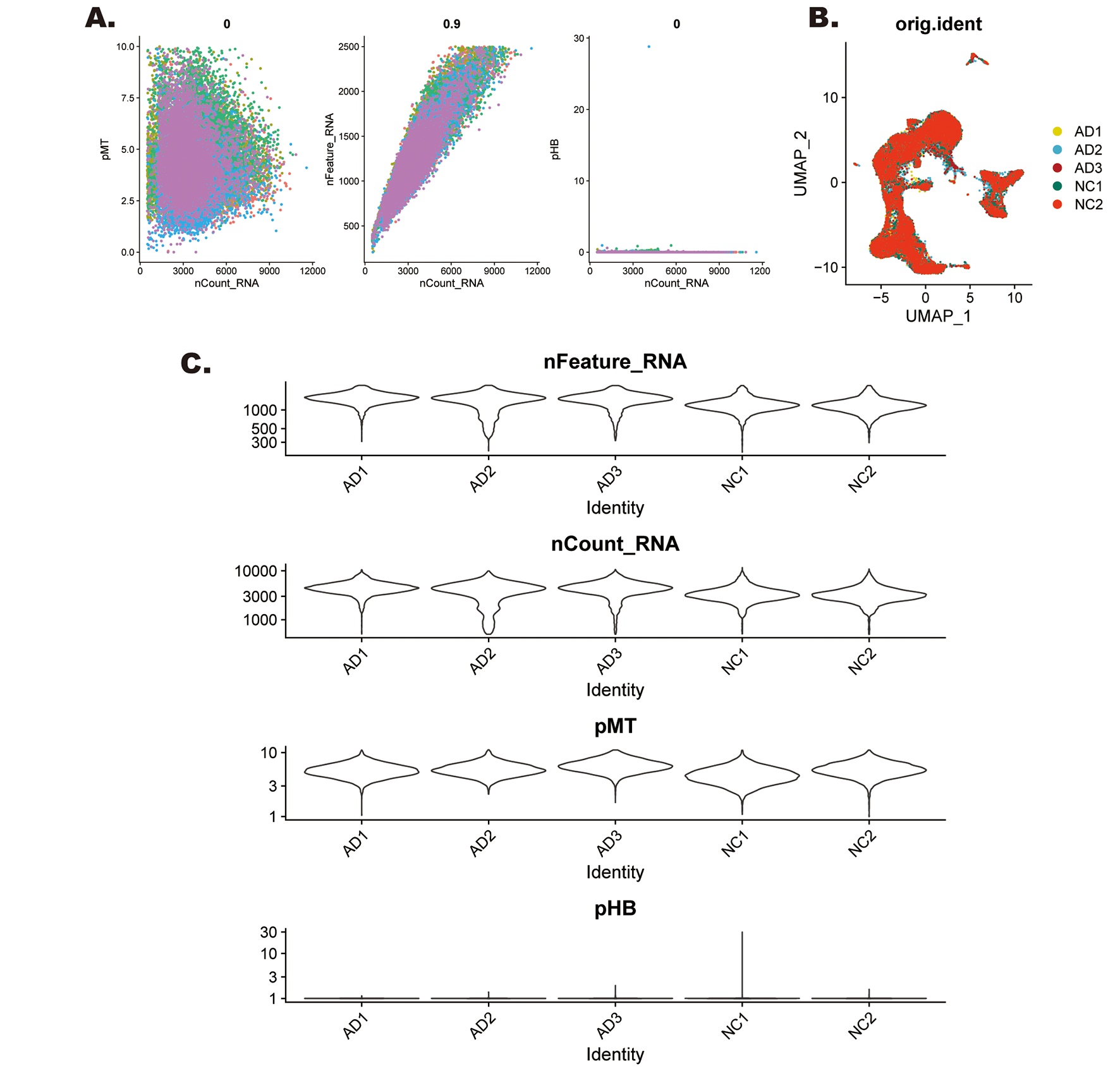

Supplement: Supplementary Figure 1 — Quality control for the scRNA-seq data of GSE181279 dataset. (A) The plot on the left represents a scatter plot of the number of mitochondria in individual cells and number of RNAs detected. The plot in the middle represents a scatter plot of the number of signature genes in relation to the number of RNAs detected. The plot on the right represents a scatter plot of the number of erythrocyte proteins mixed in the sample in relation to number of RNAs detected; (B) Distribution of all clusters is represented with a UMAP plot, where different colors represent different samples; (C) Differences in signature RNA expression, the total number of RNAs detected, pMT, and pHB in different clinical samples. pMT: percent of mitochondrial counts; pHB: percent of hemoglobin RNA counts. [file Image_1.TIF]

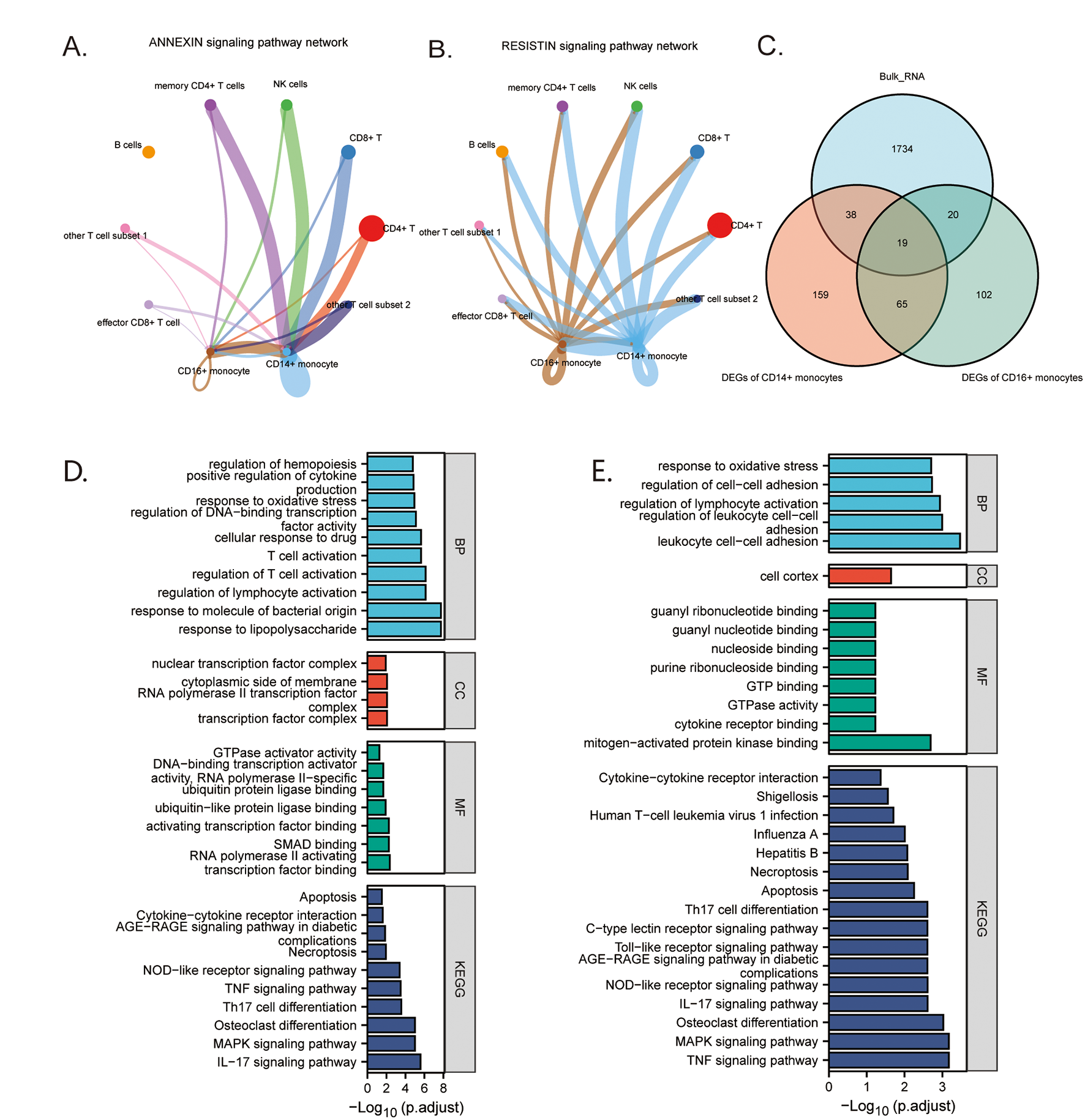

Supplement: Supplementary Figure 2 — Intersection and enrichment analysis of DEGs in CD14+ and CD16+ monocytes from Alzheimer’s Disease (AD) patients for the GSE51835 bulk RNA dataset. (A,B) Cellchat-based results demonstrating the association of specific cell populations with other cell populations on various cellular pathways, including the ANNEXIN signaling pathway (A), and RESISTIN signaling pathway (B); (C) Venn diagram showing results of intersection analysis of DEGs in CD14+ and CD16+ monocytes from AD patients for the GSE51835 bulk RNA dataset; (D) Differentially expressed genes co-expressed in CD14 + monocytes from AD patients after exercise; (E) Differentially expressed genes co-expressed in CD16 + monocytes from AD patients after exercise. [file Image_2.TIF]

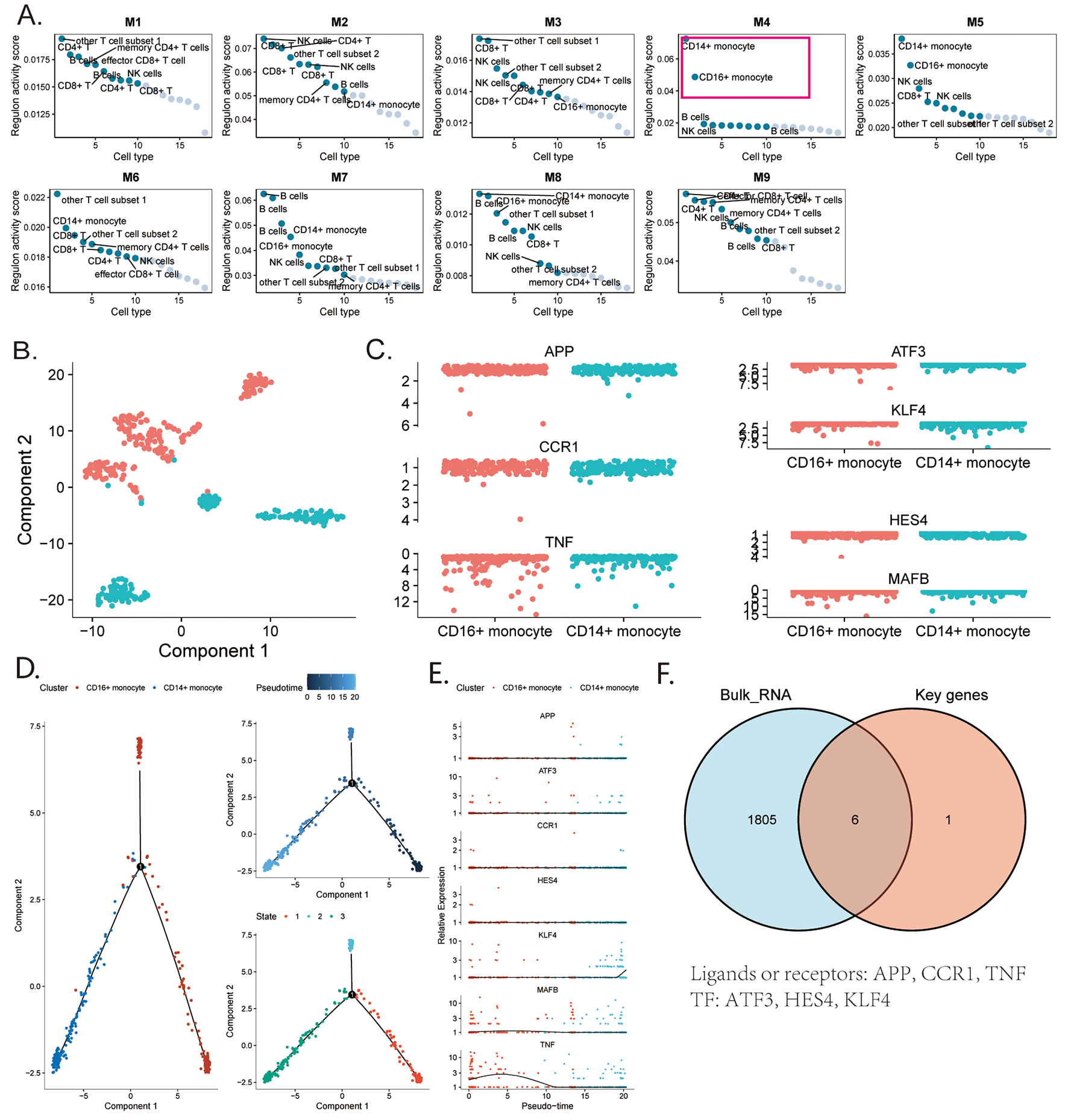

Supplement: Supplementary Figure 3 — Key transcriptome clusters of monocytes affected by exercise in AD patients with pseudotime analysis. (A) Distinct cell types’ transcriptional regulatory activity in different cell modules; (B) All CD14+ and CD16+ monocytes were subjected to a principal component analysis (PCA); (C) Scatter plots of APP, CCR1, TNF, ATF3, KLF4, HES4, and MAFB expression profiles; (D) Distribution of CD14+ and CD16+ monocytes on pseudotime trajectories, pseudotime value distribution plots, and pseudotime trajectories branching (E). APP, CCR1, TNF, ATF3, KLF4, HES4, and MAFB expression levels in relation to pseudo-time; (F) In the post-exercise bulk RNA collection, these seven important genes of APP, CCR1, TNF, ATF3, HES4, and KLF4 were discovered to be tightly related. [file Image_3.TIF]

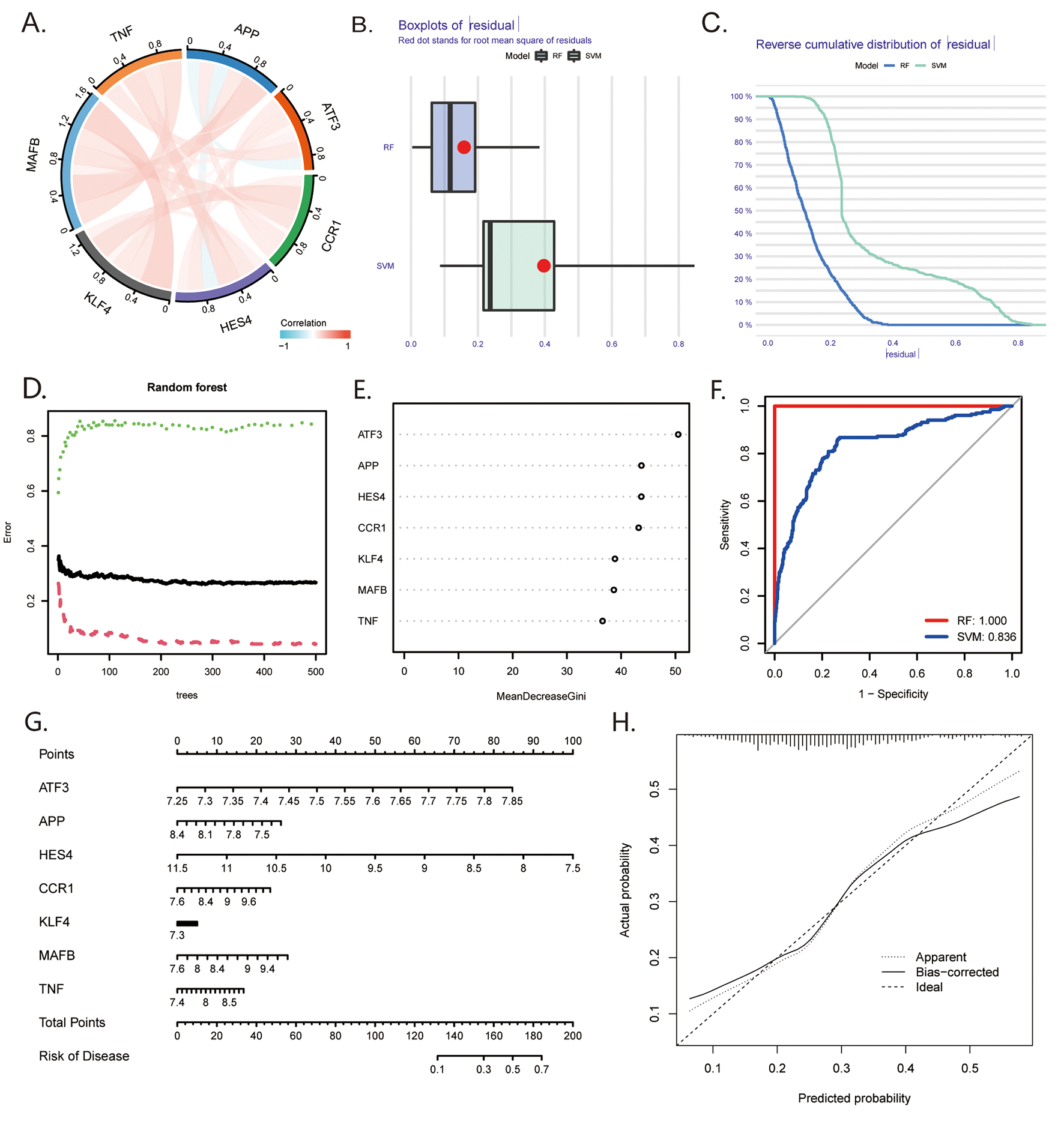

Supplement: Supplementary Figure 4 — The ERADMT gene set was found to be a potential risk marker for the development of patients with AD. (A) Correlation analysis of APP, CCR1, TNF, ATF3, KLF4, HES4, and MAFB in the GSE140831 dataset; (B,C) The box plot shows that the model residuals of SVM are larger than those of RF model; (D) Relationship between the “error” of the RF model and number of selected trees of the model; (E) Ranking of ERADMT genes according to their importance; (F) Predictive power of RF and SVM models with predictive power demonstrated by the ROC curves; (G) The nomogram prediction model constructed with the expression features of the ERADMT gene set; (H) The nomogram prediction model’s calibration curve based on the ERADMT gene set. [file Image_4.TIF]

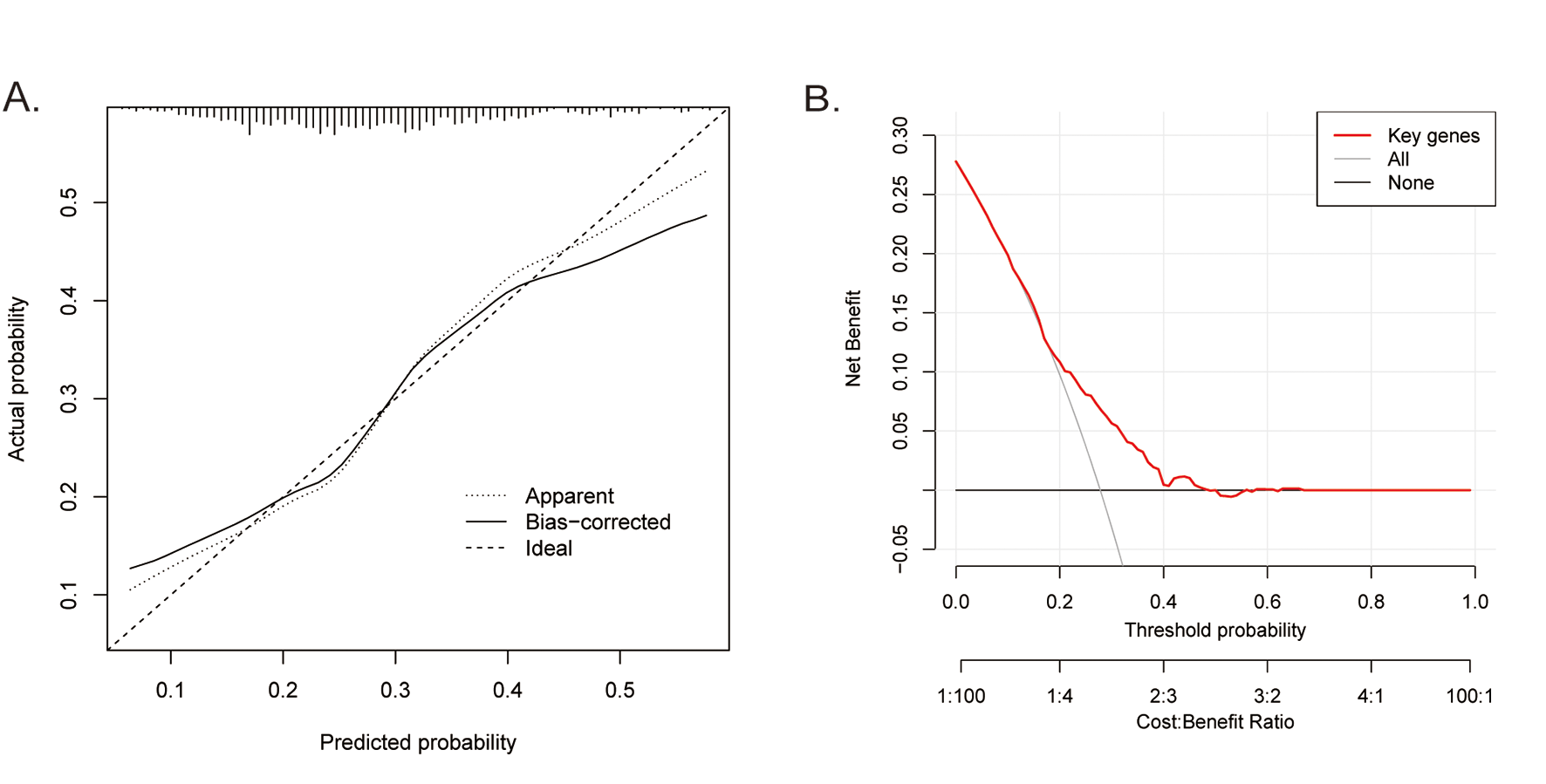

Supplement: Supplementary Figure 5 — The ERADMT gene set prediction AD risk column nomogram prediction model’s predictive power was evaluated; (A) Decision Curve Analysis (DCA) curves of the nomogram prediction model based on the ERADMT gene set; (B) The nomogram prediction model based on the ERADMT gene set underwent DCA. [file Image_5.TIF]
