# Supplementary material for: Exercise Modifies the Transcriptional Regulatory Features of Monocytes in Alzheimer’s Patients: A Multi-Omics Integration Analysis Based on Single Cell Technology
Source: Front Aging Neurosci. 2022 May 3;14:881488. doi: 10.3389/fnagi.2022.881488 (PMC9110789; doi:10.3389/fnagi.2022.881488)
Supplement: Supplementary file 7 [file Table_2.DOCX]

| Supplementary table 2. the key TFs in the M1-M9 clusters | |
| --- | --- |
| Cluster | TFs |
| M1 | ARNT, ATF1, BCLAF1, BRF2, CEBPZ, CHD2, CREB1, DNMT3A, ELK1, ELK4, FOXN2, GTF2F1, HDAC1, HDAC2, HLF, KLF9, MXI1, NFYC, NR2C1, NRF1, PATZ1, RAD21, RFX1, SETDB1, SIN3A, SMARCA4, SMARCC2, SP2, SREBF1, SUPT20H, TAF7, TIMM8A, UBTF, USF1, XRCC4, ZNF274, ZNF550, ZNF580, ZNF597, ZNF681, ZNF76, ZNF780B |
| M2 | ARNTL, BHLHE40, E2F6, EOMES, ETV1, ETV7, EZH2, HIC1, HMGB2, KLF6, MAZ, NR1D1, PRDM1, RUNX3, TBX21, TGIF1, ZBTB7A, ZMIZ1 |
| M3 | ATF2, CREB3, ELF2, ELF3, GABPA, GFI1, HCFC1, HMGN3, IRF3, IRF9, KDM5A, KLF12, MECP2, MEX3C, NELFE, NFYA, NFYB, PHF8, PML, POLE4, POLR2A, RBBP5, RELA, RXRB, SIRT6, SMAD5, SP3, SP4, TAF1, TBP, THAP1, TP53, VEZF1, ZBTB14, ZFP64, ZFX, ZNF143 |
| M4 | ATF3, BCL3, BCL6, CEBPA, CEBPD, CREB5, CUX1, E2F2, EGR1, EGR2, ETS2, ETV6, FOSB, FOSL1, FOXO3, HES4, HLX, IRF7, KLF4, KLF5, MAFB, MAFG, NFE2, NFIL3, OSR2, RARA, RCOR1, RFX2, RXRA, SRF, TFEC, USF2, ZBTB7B, ZNF467 |
| M5 | ATF5, BACH1, CREB3L2, CREM, DDIT3, ETV3, HOXA10, IRF1, ZNF787 |
| M6 | ATF6, BRF1, CEBPB, CPSF4, CTCF, E2F4, FLI1, HOXB2, HOXB3, JUND, NR2C2, RELB, REST, STAT5B, THAP11, YY1, ZNF444, ZSCAN31 |
| M7 | BCL11A, CEBPG, E2F1, E2F3, EBF1, ERF, ESRRA, HIVEP1, IRF5, IRF8, JUN, MYBL2, NFKB2, PAX5, REL, RORC, SPI1, SPIB, TBL1XR1, TCF4, TFEB, TP73, VDR |
| M8 | BHLHE41, CREB3L4, EGR4, ELF4, EP300, ETV5, GABPB1, HIF1A, IRF2, IRF4, KLF10, KLF13, MNT, PBX3, RREB1, SIX5, SNAI1, SP1, SREBF2, STAT2, TCF12, TCF3, ZBTB17, ZFHX2 |
| M9 | ELF1, ETS1, FOXP1, FOXP3, GLIS3, HINFP, IL21, JUNB, KLF16, MAF, MAFF, MAX, MYC, NR3C1, POLE3, RUNX2, STAT1, ZEB1, ZNF471 |
