# Supplementary material for: Exercise Modifies the Transcriptional Regulatory Features of Monocytes in Alzheimer’s Patients: A Multi-Omics Integration Analysis Based on Single Cell Technology
Source: Front Aging Neurosci. 2022 May 3;14:881488. doi: 10.3389/fnagi.2022.881488 (PMC9110789; doi:10.3389/fnagi.2022.881488)
Supplement: Supplementary file 8 [file Table_3.DOC]

| Supplementary table. Worksheet of public datasets | | |
| --- | --- | --- |
| Datasets | Summary | DATABASE LINK |
| GSE181279 | The nucleated cell dataset from peripheral blood in AD | <https://www.ncbi.nlm.nih.gov/geo/query/acc.cgi?acc=GSE33000> |
| GSE51835 | Mononuclear cells in peripheral circulating blood after exercise | <https://www.ncbi.nlm.nih.gov/geo/query/acc.cgi?acc=GSE44770> |
| GSE140831 | Circulating blood mRNA expression data from ADs | <https://www.ncbi.nlm.nih.gov/geo/query/acc.cgi?acc=GSE138826> |
